# Interfacial solar evaporation transforms brine mineral recovery

**DOI:** 10.1126/sciadv.adx3242

**Published:** 2025-09-19

**Authors:** Kaijie Yang, Meiqi Yang, Hongxu Chen, Sunxiang Zheng, Aashish Khandelwal, Rodney D. Priestley, Zhiyong Jason Ren

**Affiliations:** ^1^ Department of Environmental Science, Zhejiang University, Hangzhou, Zhejiang 310058, China.; ^2^ State Key Laboratory of Soil Pollution Control and Safety, Zhejiang University, Hangzhou, Zhejiang 310058, China.; ^3^ Department of Civil and Environmental Engineering, Princeton University, Princeton, NJ, USA.; ^4^ Andlinger Center for Energy and the Environment, Princeton University, Princeton, NJ, USA.; ^5^ Department of Chemical and Biological Engineering, Princeton University, Princeton, NJ, USA.

## Abstract

Interfacial solar evaporation (ISE), primarily developed for desalination, offers a potential pathway for brine mining. This perspective review provides an initial assessment of how ISE might mitigate the drawbacks of traditional evaporation ponds, such as prolonged processing and extensive land use, in the context of rising mineral demand. We examine the latest advancements in the ISE technology, focusing on materials innovations and heat management designed for high-salinity brines, which are key to enabling rapid evaporation and selective mineral recovery. Looking forward, we analyze the critical challenges that hinder the transition of ISE from laboratory to industry, including material stability, long-term durability, technoeconomic viability, and potential environmental risks. Acknowledging that the practical implementation of ISE is not yet certain, this study outlines a potential road map to guide the efforts needed to overcome these barriers and realize the goal of efficient, sustainable mineral extraction.

## Teaser

Interfacial solar evaporation offers a promising approach to more sustainable mineral mining.

## INTRODUCTION

Interfacial solar evaporation (ISE) is a photothermal process that concentrates solar energy at the liquid-air interface to enhance evaporation efficiency and minimizes heat loss. The ISE technology emerged as a promising approach for desalination, and it has undergone a remarkable evolution in recent years. Leveraging state-of-the-art photothermal conversion materials (*1*–*5*), advanced heat management strategies (*6*–*15*), and innovations in evaporation enthalpy reduction (*16*–*18*), the solar-to-vapor efficiency has approached nearly 100%. Moreover, by harnessing additional environment energy (*19*–*22*), such as wind and ambient heat, some ISE systems (*22*, *23*) have demonstrated evaporation rates as high as ~10 kg m^−2^ hour^−1^, several times the theoretical solar evaporation limit under 1 sun. Researchers have also developed robust structural designs that mitigate salt crystallization and accumulation, ensuring stable, long-term operation. The development of ISE technology has been extensively documented in recent review articles (*24*–*37*) that this study will not repeat. However, what we want to highlight in this study is that ISE applications remain narrowly focused on seawater desalination and wastewater treatment, overlooking its notable potential in other fields requiring fast evaporation.

One such opportunity lies in brine mining, where the escalating demand for critical minerals like lithium presents a new opportunity for ISE (*38*). As electric vehicles and renewable energy storage systems proliferate worldwide, the demand for lithium is expected to surge from 0.5 million metric tons of lithium carbonate (Li_2_CO_3_) equivalent (LCE) in 2021 to more than 3 million tons by 2030 (*38*, *39*). Brine represents a critical mineral resource, enriched with a diverse array of elements (*40*–*43*), including but beyond Li^+^. Brine hosts a complex mixture of cations such as sodium (Na^+^), potassium (K^+^), calcium (Ca^2+^), and magnesium (Mg^2+^), alongside anions including chloride (Cl^−^), bromate (BrO_3_^−^), nitrate (NO_3_^−^), carbonate (CO_3_^2−^), and sulfate (SO_4_^2−^). This multifaceted composition positions brine as a versatile feedstock with substantial potential for a broad range of high-value applications, spanning energy storage, agriculture, and advanced material synthesis. Now, nearly all industrial brine extraction processes depend on conventional solar evaporation ponds. Although this approach is straightforward and widely adopted, it poses notable environmental, social, and economic challenges, particularly in sensitive regions (*44*). For example, in Chile’s Atacama Desert—one of the driest places on Earth—these ponds occupy ~30 km^2^ (an area more than twice the size of Lower Manhattan) and require 15 to 18 months to complete one mineral concentration and extraction cycle (*45*). Such operation was reported to contribute to land subsidence and destabilize local ecosystems and infrastructure. Moreover, the loss of natural habitats affects wildlife such as the Andean flamingo (*46*), while intensified competition for limited land and resources has sparked conflicts with local communities regarding the long-term sustainability of their traditional livelihoods (*38*, *47*, *48*).

The confluence of escalating global demand for minerals and growing environmental concerns over current evaporation pond practices underscores a pivotal opportunity for the integration of ISE into brine mining. The ISE technology has the potential to transform brine mining by accelerating evaporation rates while reducing the spatial footprint, ecological impact, and chemical usage of traditional processes. This innovation not only boosts throughput but also supports more sustainable and efficient resource recovery, aligning with broader goals of environmental stewardship. Accordingly, this perspective review explores the transformative potential of ISE in brine mining, emphasizing its capacity to redefine efficiency, environmental stewardship, and economic viability. It begins by analyzing the limitations of conventional evaporation-based mineral extraction, followed by an overview of advanced salt-tolerant solar evaporators tailored for brine environments. The review then examines the integration of ISE technologies into practical applications, highlighting their ability to accelerate evaporation, enable chemical-free separation, and facilitate water harvesting. Last, it addresses the challenges and prospects of scaling ISE strategies from lab research to industrial implementation, aiming to inspire innovative approaches and advance sustainable brine resource extraction. This perspective aims to foster dialog, inspire innovation, and advance the discourse on sustainable strategies for resource extraction from brine.

## CURRENT PRACTICES AND CHALLENGES OF BRINE MINING BY SOLAR EVAPORATION PONDS

**Fig. 1.**
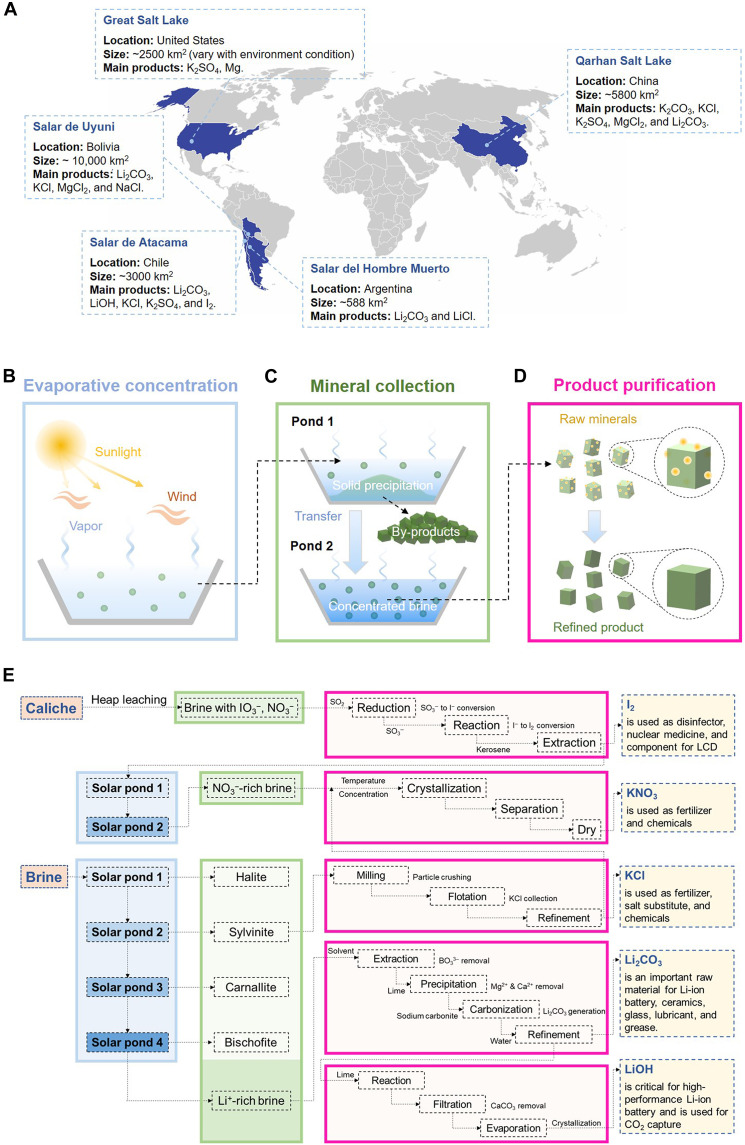
Global brine distribution and solar evaporation–based mineral recovery. (**A**) Global distribution of major brine resources, highlighting their characteristics of geographical distribution, Salar size, and key products. (**B**) Schematic representation of the evaporative concentration process. (**C**) Demonstration of the step-by-step mineral collection process during brine concentration. (**D**) Illustration of final product refinement after the initial mineral isolation. (**E**) Representative evaporative mining workflow depicting the conversion of raw brine to target products. LCD, liquid-crystal display.

Brine resources are distributed globally, with substantial reserves concentrated in regions such as Chile, Argentina, and Bolivia in South America, an area known as “Lithium Triangle.” China and North America also have brine sources but with lower mineral concentrations (Fig. 1A). Because of the differing geological, climatic, and hydrological conditions of each region, no two brines share the same composition. Brine mining technologies are highly specialized across regions to address the distinct characteristics of each deposit (*49*). Despite this variability, evaporation ponds remain a cornerstone of numerous industrial processes, with tens of thousands of hectares in operation globally. In the salt flats of Chile and Argentina, these ponds are primarily used for lithium recovery and potash extraction. In the United States, they are used for potash and magnesium production, while in China, they play a crucial role in the recovery of sodium, potassium, and magnesium salts. The enduring reliance on evaporation ponds arises from their simplicity, cost-effectiveness, and scalability. A typical evaporation-based mining process can be divided into three stages: evaporative concentration (Fig. 1B), mineral collection (including solid precipitation and concentrated brine) (Fig. 1C), and product purification (Fig. 1D). Throughout the stages, fractional crystallization plays a pivotal role to sequentially remove undesired salts and enrich target minerals in brine systems.

Brine is a chemically complex solution containing a variety of dissolved salts, among which high-value minerals such as lithium are often present in low concentrations. To efficiently recover these high-value components, fractional crystallization is strategically used to remove nontarget salts before downstream extraction. The crystallization sequence is thermodynamically governed by the solubility and concentration of each salt component. Typically, along with evaporation, salts such as NaCl crystallize first because of high concentration and low solubility, followed by others in a defined order. This process is highly sensitive to temperature and ionic composition, both of which notably influence phase equilibria. For instance, higher temperatures generally increase salt solubility, while changes in ionic composition shift crystallization thresholds. To predict and navigate the crystallization sequence, phase diagrams and thermochemical modeling are extensively used, while experimental approaches such as isothermal dissolution and evaporation methods provide critical phase data to understand both stable and metastable phase behavior in complex brine systems (*50*). In industrial settings, engineered crystallizers are widely deployed to regulate the crystallization pathways. Compared to solar ponds that rely on fluctuating environment, engineered crystallizers allow for fine-tuned control over key parameters such as temperature and stirring rate, thereby enabling targeted and efficient crystallization of specific salts (*51*, *52*). However, this level of control typically requires substantial energy input for heating, cooling, and mechanical operation, leading to higher operational costs relative to passive solar evaporation systems.

Taking Sociedad Química y Minera de Chile S.A. (SQM) as an example (Fig. 1E), the company is the world’s largest producer of brine lithium, as well as a leading producer of iodine and potassium nitrate. Its mining process is highly integrated with evaporation throughout the entire operation. For iodine and nitrate, the extraction process begins with heap leaching of solid caliche ore, producing brine rich in IO_3_^−^ and NO_3_^−^. Iodine is recovered from this brine through reduction with SO_2_ followed by kerosene extraction. Subsequently, nitrate in the brine undergoes evaporative concentration in sequential solar ponds, where crystallization is achieved through precise temperature control. For potassium and lithium, the operation starts by pumping brine into large, shallow evaporation ponds, where it is exposed to sunlight for evaporative concentration. As salinity rises, the brine is transferred through a series of ponds to control precipitation conditions for targeted mineral extraction. During this fractional crystallization process, halite (NaCl) typically precipitates first, followed by sylvinite (a mixture of NaCl and KCl) and then bischofite (MgCl_2_·6H_2_O) as a by-product. This multistage evaporation process ultimately yields lithium-rich brine with a concentration of ~6% Li^+^ as well as other solid minerals along the way (*45*). Concentrated LiCl solutions are transported to refineries, where they undergo chemical processing to produce Li_2_CO_3_ or LiOH for various applications. For Li_2_CO_3_ production, lithium-rich brine first undergoes solvent extraction and selective precipitation to remove impurities followed by carbonization and water washing. If LiOH is desired, the obtained Li_2_CO_3_ undergoes further reaction with lime (CaO), followed by crystallization to yield the final product. By-product solid minerals, such as potassium chloride (KCl), are further refined through milling, flotation, and refinement to meet market specifications for use in industries like agriculture and chemical manufacturing.

While solar evaporation ponds are a cost-effective method for mineral extraction and processing, they have notable limitations. The inherently slow evaporation rate extends production times and requires vast areas of land to meet production demands, which pose environmental and socioeconomic challenges, particularly in fragile ecosystems. While the evaporation process effectively isolates minerals, subsequent purification stages often rely on intensive chemical usage, increasing process complexity and environmental impact. These challenges highlight the urgent need for innovative approaches to enhance the efficiency of evaporation-based mining, streamline operations, and reduce environmental footprint.

## HIGH-SALINITY BRINE PRESENTS OPPORTUNITIES AND CHALLENGES FOR ISE DESIGN

**Fig. 2.**
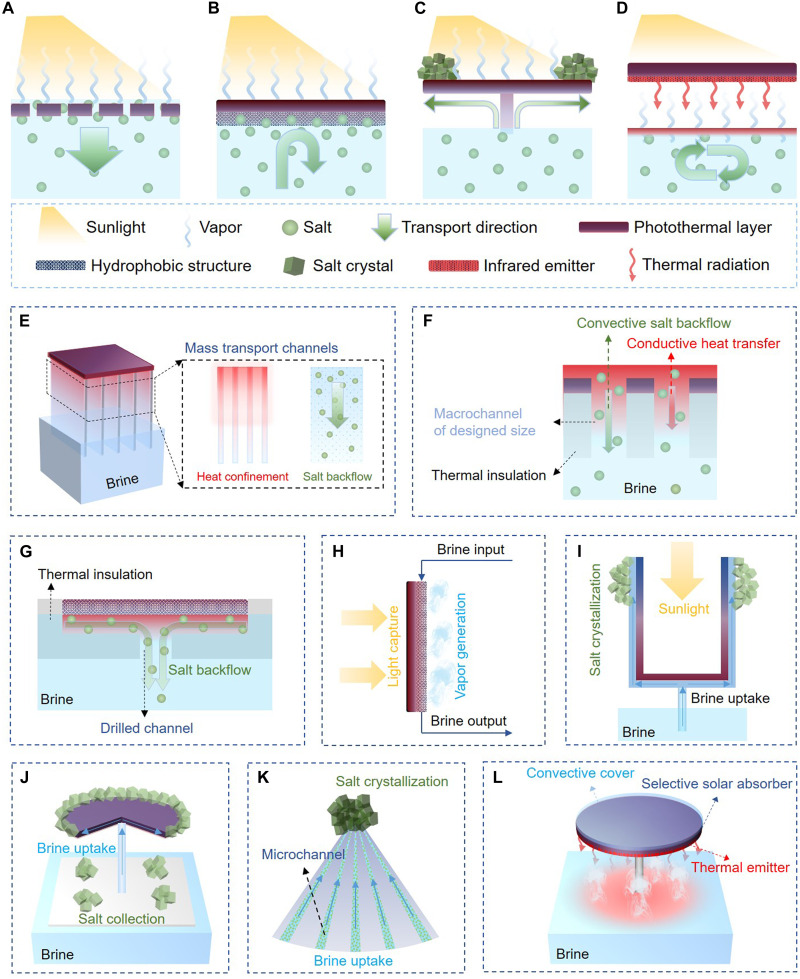
Design strategies of salt-tolerant solar evaporators. (**A**) Schematic of salt backflow enhancement to prevent salt accumulation. (**B**) Illustration of Janus structure design to avoid brine insulation. (**C**) Demonstration of directional salt crystallization via specific architecture design. (**D**) Contactless design to avoid salt fouling. (**E**) 3D evaporator design with mass transport bridges for efficient salt backflow. (**F**) The evaporator design relies on fluid flow optimization to enable efficient salt backflow and heat management. (**G**) Water lily–inspired Janus evaporator designed for salt rejection and heat confinement. (**H**) Vertical Janus structure to avoid salt accumulation by positioning the photothermal layer and brine transport layer on opposite sides. (**I**) Cup-shaped solar evaporator with salt cyclized on the cup outside wall. (**J**) Salt-resistant evaporator designed for automatic salt harvesting, with salt crystallized at the edge. (**K**) Cone-shaped evaporator featuring localized crystallization at the apex. (**L**) Umbrella-shaped, contactless evaporator designed for high-salinity brines.

The Salar brine exhibits salinity levels ranging from 30 to 40% (*44*, *53*), making it ~8 to 12 times more saline than seawater. This extreme salinity results in a highly corrosive medium that greatly accelerates material degradation and fouling, posing substantial challenges of durability and operational efficiency of any devices for extraction and processing. While the ISE technology is highly efficient in capturing sunlight, converting it into heat energy, and maximizing heat utilization for vapor generation, most ISE devices designed for desalination and freshwater production are not directly translatable to brine mining applications. In such high-salinity environments, substantial salt accumulation rapidly occurs, quickly reducing light capture, blocking vapor release channels, and causing rapid failure. Our experience in using traditional designs of ISE reported in the literature has shown that these devices can sink and fail within just a few days under brine conditions. Therefore, salt-tolerant or salt-resistant designs are essential for guiding evaporator design for brine mining. This study evaluates the evolution of these designs over the past decade with a particular focus on representative systems suitable for high-salinity (≥20 wt %) or saturated brine environments, as well as designs that enable directional crystallization to facilitate mineral recovery. We highlight several advanced design strategies, including salt backflow enhancement (Fig. 2A), Janus structural design (Fig. 2B), directional crystallization (Fig. 2C), and contactless configuration (Fig. 2D). These approaches demonstrate promising potential for overcoming salt-related challenges and improving the robustness of ISE devices for brine mining applications.

### Salt backflow enhancement

Salt backflow is a straightforward approach to reverse the salt accumulation. As the evaporation processes, the salinity at the evaporative interface gradually increases, creating a concentration gradient within the evaporator. This gradient, coupled with the associated density differences, drives the spontaneous movement of excess salt away from the interface and back into the bulk brine (Fig. 2A). The process relies on natural diffusion and convection mechanisms to redistribute the salt, preventing its buildup and maintaining the functionality of the evaporative surface. The backflow flux ( J ) can be described by the diffusion-convection equation (*54*, *55*) $J=\varepsilon A\left(D_{diff}\nabla C/L+D_{\text{convec}}\nabla \rho \right)$ , where ε and A represent the porosity and the cross-sectional area of the mass transport structure, respectively. L is the length of the transport path. $D_{\text{diff}}$ and $D_{\text{convec}}$ are the diffusion and convection coefficients of salt, while ∇C and ∇ρ represent the concentration and density differences, respectively.

To avoid salt crystallization, the rate of salt backflow must keep pace with the rate of salinity increase, which necessitates the evaporator to be equipped with efficient channels to facilitate the mass transport of salts. Innovative strategies to enhance the salt backflow include creating a hydrophilic porous structure (i.e., increasing ε ) (*56*–*58*), increasing channels for mass transport (i.e., improving A ) (*59*–*61*), and designing vertical or wick-free channels (*62*–*65*) to minimize tortuosity (i.e., reducing L ). These methods have been proven to effectively enhance the salt-tolerant capacity of the evaporator, ensuring stable performance in seawater or even brines with salinity as high as 20% (*56*, *60*–*62*, *64*, *66*). In addition to diffusion and convection, another well-known phenomenon is the Marangoni effect (*67*, *68*), which is a result from the surface tension gradient induced by concentration and temperature differences. This effect is driven by the surface tension difference that can move liquid from a low-surface-tension region to those with high surface tension, achieving efficient liquid transfer. Diffusion, convection, and the Marangoni effect are driven by different principles, but they are often interwoven and collectively contribute to salt backflow.

While enhancing the salt backflow, special attention must be given to minimizing parasitic heat dissipation, as rapid salt backflow often carries heat energy away from the photothermal layer and results in low evaporation efficiency (*55*, *63*, *64*, *69*). To prevent heat dissipation, mass transport structures that separate the photothermal layer from the bulk water have proven to be an effective solution (*55*, *63*). In these designs, the photothermal layer is typically connected to the bulk water through mass transport structures, which not only ensure efficient water uptake and salt backflow but also recycle the heat conducting along the structure for evaporation, thus minimizing heat dissipation into the bulk water (Fig. 2E). Optimizing the fluid flow is another effective approach to mitigate parasitic heat loss caused by salt backflow. This strategy leverages the substantial difference between salt diffusivity (~10^−9^ m^2^ s^−1^) and heat diffusivity (~10^−7^ m^2^ s^−1^) to control mass and heat transfer. It allows salt to backflow via rapid convection, while heat is transferred via a relatively slow diffusion process (Fig. 2F). Zhang *et al.* (*64*) demonstrated that using a wick-free structure to control fluid flow can achieve a solar-to-vapor efficiency over 80% in 20 wt % salinity brine. Salt backflow has expanded the potential for solar evaporators to operate in brines. However, it is important to recognize that the driving force relies on concentration and density differentials (i.e., ∇C and ∇ρ ). As a result, the effectiveness of this approach diminishes in high-salinity or near-saturated brines, necessitating the development of other alternative salt-tolerant strategies.

### Janus structure design

Janus structure design is another effective strategy to prevent surface salt accumulation. The principle of this design lies in preventing brine intrusion into the photothermal structure by decoupling the photothermal layer from the brine supply structure (Fig. 2B). Most Janus structures comprise two functional components: an upper photothermal structure for light-to-heat conversion and lower water-wicking structure for brine supply, confining the evaporative layer to the interface between the two layers (*70*, *71*). To facilitate stable and efficient evaporation, the photothermal structure is porous to allow vapor release and hydrophobic to prevent brine intrusion. Recent advancements have introduced innovative Janus structures designed for brine environments, such as electrospun flexible absorbers (*70*), Janus MXene aerogels (*72*), and silica nanofibrous aerogels (*73*). Some specialized evaporators can operate effectively in brines with salinities exceeding 20 wt % (*71*).

Beyond salt-rejection capability, an outstanding Janus evaporator must also incorporate effective thermal management. This is critical because the photothermal layer in a Janus structure is often in close contact with the bulk brine, leading to undesirable heat dissipation. To address this challenge, design strategies focus on integrating thermal insulation structures that minimize heat loss while ensuring efficient water supply and salt rejection (*71*, *74*). Taking the water lily–inspired hierarchical evaporator as an example (Fig. 2G) (*71*), it features a hydrophobic porous solar absorber in the upper section and a perforated polystyrene bottom stand (thermal conductivity <0.04 W m^−1^ K) in the lower section. The perforated polystyrene base not only facilitates water and salt transfer but also provides excellent thermal insulation, notably reducing heat loss. Such a design enables stable operation in brine with ~30 wt % salinity, achieving an evaporation rate of 1.27 kg m^−2^ hour^−1^. In addition to the structures based on porous hydrophobic photothermal layers, innovative designs such as ion-selective hydrogels (*75*) have emerged. Such structures facilitate ion-electromigration salt removal through the combination of cationic and anionic hydrogels. Another advancement is the vertical Janus evaporator, which strategically positions the photothermal layer and evaporative layer onto opposite sides of an impermeable barrier (Fig. 2H) (*76*), preventing the photothermal side from contacting the brine. These advancements offer unique insights for advancing solar evaporation in high-salinity brines.

### Directional crystallization

In contrast to designs that seek to prevent salt accumulation, directional crystallization revolves around meticulous control of the crystallization sites to minimize the adverse effects of salt accumulation (Fig. 2C) (*77*–*81*). Through carefully engineered structures, this strategy can even enable the automatic detachment of crystals, facilitating salt recovery (*78*, *80*, *82*). At the onset of evaporation, the brine concentration is uniform within the evaporator. As evaporation progresses, fresh brine replenishes the released water. However, because of variations in salt backflow flux across different areas, a salinity gradient begins to form within the evaporator. Once specific regions reach saturation, crystallization initiates in these high-salinity zones. Achieving controllable crystallization hinges on managing the mass transfer of water and ions within the evaporator to ensure that salt crystallizes at the desired location. Through innovative structural engineering aimed at optimizing salinity distribution, effective rational structures have been developed. Examples include cup-shaped crystallizers (*77*, *79*), umbrella-shaped structures (*78*, *83*–*85*), conical evaporators (*82*), and inclined evaporation surface (*86*–*88*). Each of these designs facilitates crystallization at designated locations, such as the sidewalls (Fig. 2I) (*77*, *79*), edges (Fig. 2J) (*78*, *83*–*88*), and structure apex (Fig. 2K) (*82*), effectively minimizing the impact of salt accumulation on photothermal conversion.

Furthermore, reducing the adhesion of crystallized salt to the evaporator can facilitate the automatic detachment of the salt, promoting their recovery. Studies have shown that increasing the water supply to rewet the contact points between salt and the evaporator is an effective approach for improving the detachment process (*78*, *86*). Meanwhile, harnessing environmental energy, such as wind, can greatly improve the salt recovery performance (*22*), as the crystallization speed is closely linked to the evaporation rate. However, it is crucial to recognize that while some evaporators may function effectively in NaCl solutions, they often fail when handling real brines that contain a variety of ions beyond NaCl, such as Mg^2+^, Ca^2+^, and SO_4_^2−^ (*79*, *83*, *85*). Although these ions are present at much lower concentrations than Na^+^, they can substantially affect the crystallization process. NaCl crystals typically form discrete cubic structures with loose packing, where minor accumulation does not substantially affect the evaporators’ performance. However, when Mg^2+^, Ca^2+^, and SO_4_^2−^ coexist, CaSO_4_ crystals tend to adhere on the surface of NaCl crystals, while MgSO_4_ fills the gap between them (*79*). This results in a dense, compact structure that clogs the pore structure of the evaporator, ultimately reducing its efficiency and shortening its lifespan. Although the use of anticrystallization agents can help mitigate the dense salt accumulation (*79*, *85*), it involves notable chemical consumption when scaling up, and the complex crystallization process warrants further attention.

### Contactless evaporation

Different from the strategies that rely on conduction to transfer heat from the photothermal layer to brine, contactless configuration uses thermal radiation to facilitate heat transfer and therefore enhance evaporation efficiency. This strategy establishes the spatial separation of the photothermal layer and the brine, thus fundamentally eliminating the influence of salt fouling on the photothermal structure. As demonstrated in Fig. 2D, a typical contactless configuration consists of a selective solar absorber designed for photothermal conversion while minimizing thermal radiation loss. Beneath the solar absorber, an infrared emitter is closely attached, drawing heat from the solar absorber and radiating it downward in infrared radiation. Water exhibits strong absorption in the infrared wavelength (absorption coefficient of ~10^4^ m^−1^) (*89*), effectively confining radiative heat within the top 100-μm-thick water layer, thereby driving rapid interfacial evaporation. This contactless configuration eliminates the impact of salinity on system stability, demonstrating very good potential for evaporating saturated brines. For instance, a contactless sun umbrella (Fig. 2L) has shown double the evaporation rate of saturated NaCl solutions (*89*). Moreover, benefiting from the thermal separation between the solar absorber and the bulk solution, this contactless setup can even generate steam at temperatures up to 133°C under 1-sun illumination (*90*).

The challenges posed by high-salinity brines, such as accelerated material degradation, salt accumulation, and heat dissipation, underscore the need for innovative ISE designs tailored for extreme environments. The evolution of salt-tolerant and salt-resistant strategies, including salt backflow enhancement, Janus structural designs, directional crystallization, and contactless configurations, each addresses critical limitations of traditional ISE devices. These advanced designs mitigate salt accumulation but also improve thermal management, operational stability, and efficiency, even in near-saturated brines. However, many challenges remain as further advancements are needed to address complex multicomponent brines containing ions like Mg^2+^, Ca^2+^, and SO_4_^2−^, which exacerbate crystallization and clogging issues. Also, the evaporation by Janus and contactless designs during nighttime may be reduced because of the additional vapor transport resistance to the natural evaporation process. Nearly all these devices have been demonstrated exclusively at the lab scale, predominantly using synthetic brines. Most studies focus on desalination, meaning that the tested conditions do not accurately reflect the harsh environments and challenges associated with real brine operations for mineral extraction.

### Intregrating ISE for more efficient and sustainable brine mining

**Fig. 3.**
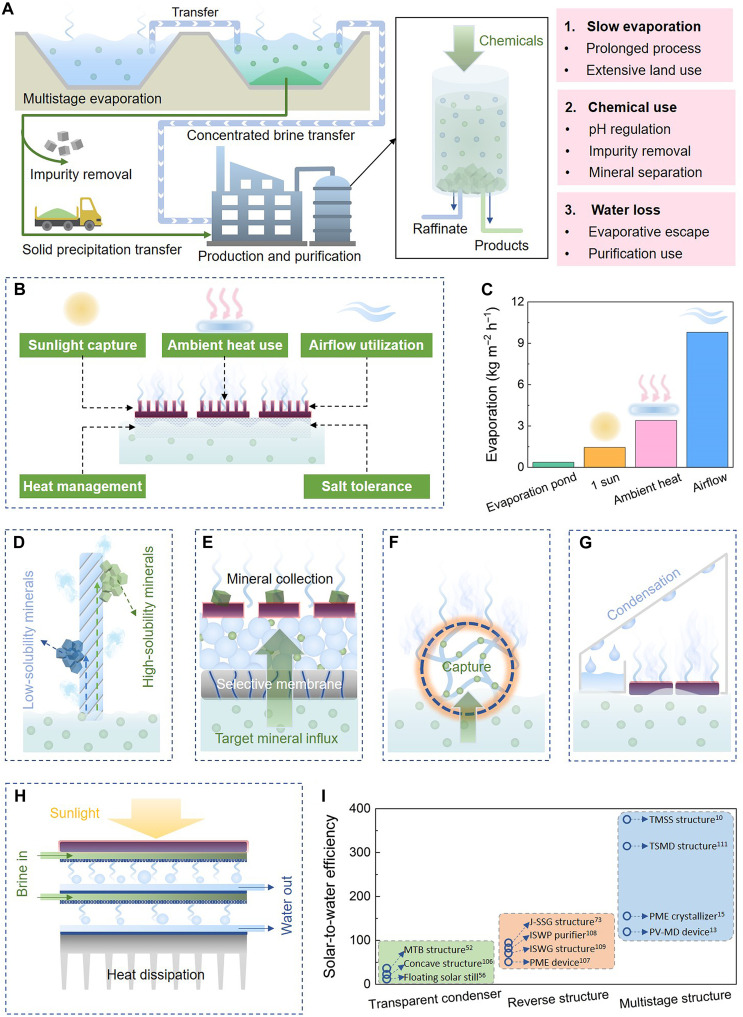
Approaches for integrating ISE with brine pond operation. (**A**) Schematic of the brine mining process with highlighted challenges of slow evaporation, intensive chemical use, and substantial water loss. (**B**) Illustration of the ISE technology that enhances evaporation through efficient sunlight capture, ambient heat use, convective airflow utilization, heat management, and salt-tolerant structure design. (**C**) Evaporation rate comparison of evaporation pond, theoretical limit of ISE (under 1 sun), and a string evaporator harnessing ambient heat and convective airflow (at a 1.5 m s^−1^ wind speed) (*39*). h, hour. (**D**) Demonstration of a string evaporator that spatially separates minerals and increases evaporation rate. (**E**) Solar–driven device for targeted mineral extraction through selective membranes. (**F**) Demonstration of target mineral capture through selective adsorption. (**G**) Schematic of solar still for freshwater collection through vapor condensation. (**H**) Demonstration of a multistage structure for enhanced freshwater generation through latent heat recovery. (**I**) Solar-to-water efficiency comparison of typical solar stills that recover vapor using a transparent condenser, reverse structure, and multistage structure.

For nearly a century, solar evaporation ponds have been the backbone of current industrial brine mining processes, attributed to their simplicity, scalability, and low cost by taking advantage of the environmental energy (*44*, *49*, *91*). However, these traditional ponds face notable challenges in meeting the demands of modern production, which requires high efficiency, better quality, and sustainable operations that minimize harm to the surrounding environment and society (*38*, *44*, *92*). The prolonged processing times, substantial land requirements, intensive chemical use, and associated environmental concerns such as water loss and ecological disruption underscore the urgent need for technological advancements to enhance efficiency and sustainability (Fig. 3A). Integrating the ISE technology into existing ponds offers a transformative solution. ISE can accelerate evaporation rates, improve mineral recovery, and address critical issues such as freshwater conservation. By improving the efficiency, it offers the potential to reduce the need for future land expansion of solar ponds, paving the way for more sustainable and efficient brine mining operations.

### ISE for accelerated evaporation

Most continental brine reserves are situated in low-latitude, high-altitude regions (*44*), where abundant sunlight and dry climates create favorable conditions for evaporation. Despite this, traditional evaporation ponds still require 15 to 18 months to produce the final products (*39*). This time-consuming operation is primarily due to the limited evaporation rate of conventional pond systems (Fig. 3A). One key factor is the inherently low light absorption coefficient of water (~10^−2^ m^−1^) (*93*, *94*). With the typical pond depth ranging from 0.5 to 2.0 m (*95*), much of the solar energy penetrates and disperses throughout the bulk water rather than being efficiently used for evaporation. While certain dissolved minerals can enhance light absorption, only about 50% of the incoming solar radiation effectively contributes to evaporation in traditional systems (*34*, *96*). This already limited light utilization is further compromised by biological interference. Over prolonged operation, algal overgrowth frequently occurs in open-air ponds, scattering and reflecting incident sunlight and thereby hindering light capture. A common mitigation strategy involves the periodic application of algaecides, such as copper sulfate (CuSO_4_) (*97*). However, the continued input of Cu^2+^ raises concerns about ecological toxicity (*98*). On the other side, some sites cultivate pigment-rich halotolerant bacteria to darken the brine and enhance light capture (*99*), but excessive biomass can elevate organic loading, disrupt crystallization dynamics, and compromise the purity of the final mineral products. In addition to light utilization, wind plays a crucial role in accelerating vapor release (*92*), but the limited surface area of solar ponds, where only the exposed surface is used, restricts the optimal use of airflow’s kinetic energy, limiting evaporation efficiency. These limitations not only result in prolonged production cycles and extensive land requirements but also lead to reduced adaptability to market fluctuations and limited profitability.

To overcome these limitations, the ISE technology offers great potential to enhance evaporation rate by localizing heat at the water-air interface, directly driving the phase change of water through latent heat conversion rather than being dissipated into the bulk water as less effective sensible heat (Fig. 3B). Central to the ISE technology is the use of photothermal materials, which are engineered to optimize sunlight utilization. Through nanostructure engineering, advanced photothermal materials can achieve light absorption exceeding 90% (*24*, *30*, *34*), with some cutting-edge designs achieving nearly 100%, more than double the efficiency of traditional ponds. For example, the carbonized wood demonstrates an impressive light absorbance of 99% across the entire solar spectrum (*100*). Rapid evaporation requires not only efficient light capture but also the direct use of solar energy for water phase change, minimizing losses to bulk water heating or the environment. Effective thermal management, such as using low-thermal conductivity materials [e.g., polystyrene foam (*7*) and porous carbon foam (*6*)] and optimizing water channels (*8*, *101*), reduces conductive heat loss. Moreover, using selective solar absorbers and convection covers can further reduce radiative and convective heat exchange between the evaporator and surroundings, minimizing the heat dissipation to the environment. These heat management strategies drive solar-to-vapor efficiency close to 100%, pushing the evaporation rate toward the theoretical limit (i.e., ~1.45 kg m^−2^ hour^−1^ under 1 sun). A first field demonstration of an ISE product called Lilypad in Chile observed a 40 to 122% increase in evaporation rates compared to the control ponds, and the extent of improvement varied depending on the type of brine treated (*102*). Integration of these photothermal materials into evaporation ponds presents a viable solution to improve the pond efficiency.

Beyond optimizing sunlight utilization, the ISE technology can further boost evaporation rate by harnessing environmental energy sources, such as wind and ambient heat, through three-dimensional (3D) structure design. These 3D architectures leverage capillary forces to draw brine upward, effectively expanding the evaporation area vertically, which accelerates evaporation rates without requiring additional land (*20*, *39*, *103*). For instance, a vertical fiber-based evaporator achieved an impressive evaporation rate of ~10 kg m^−2^ hour^−1^ under 1-sun illumination (*39*), which is ~6.9 times the theoretical evaporation limit. This remarkable increase is largely attributed to the cooler evaporation surface, which harnesses the ambient heat. Furthermore, the 3D architecture provides an expanded contact area that further accelerates convective airflow to enhance evaporation. At a wind speed of 1.5 m s^−1^, the evaporation rate of this fiber-based evaporator can increase markedly to 9.8 kg m^−2^ hour^−1^. In comparison, traditional solar ponds have annual evaporation rates equivalent to 0.11 to 0.46 kg m^−2^ hour^−1^ (*45*), so incorporating such designs into the ponds could potentially reduce pond processing times by up to 20 times, from 15 months to 3 weeks (Fig. 3C). Notably, ISE technologies can be seamlessly integrated into existing solar ponds to enhance evaporation rates, thereby improving mining efficiency and avoiding further land occupation. This inherent compatibility gives ISE a distinct advantage in upgrading existing solar pond systems toward more sustainable and land-efficient lithium production.

### ISE for enhanced mineral recovery

Mineral recovery is the primary objective of brine mining, and precipitation occurs only when the brine concentration reaches saturation, which takes a long time before harvesting. In addition, previous studies report that 10 to 15% of the minerals can be lost because of leakage beneath the pond, highlighting inefficiencies in traditional pond systems (*48*). Meanwhile, the buildup of precipitations in the ponds necessitates periodic maintenance to transfer the deposits (*45*). These maintenance operations often require halting production for extended periods, ranging from a few days to several weeks (Fig. 3A). Such interruptions increase operation complexity, diminish efficiency, and also pose risks to sustainability. Faster evaporation shortens the harvesting cycle, reduces the leakage period, and improves the overall mineral recovery, addressing some key limitations and enhancing the efficiency of brine mining operations.

Evaporators with directional crystallization capabilities are believed to hold good potential in overcoming the issues related to mineral collection. As detailed in the “Directional crystallization” section, these specially designed evaporators enable the precise control of salt crystallization sites within the system. This innovation facilitates the automatic detachment and recovery of the grown crystals, thereby accelerating evaporation and enabling continuous and automatic mineral recovery. Integrating these evaporators with evaporation ponds could notably streamline the operation and enhance the overall efficiency, potentially paving the way for maintenance-free operations. In addition, the evaporators condition the brine chemistry by maintaining lower and stable pond temperatures, which improve crystallization dynamics and selective precipitation. For example, the large-scale deployment of the Lilypad evaporator has been shown to alter the thermal profile of the pond. By localizing heat at the surface, Lilypad evaporators increase the pond surface temperature by 50 to 150% while simultaneously reducing the pond bottom temperature by 5° to 10°C. This heat management not only enhances evaporation but also promotes the precipitation of sodium chloride and astrakanite, improving the overall mineral extraction efficiency (*104*).

Once minerals are collected, either as solids or concentrated liquids, they undergo a series of purification steps to obtain the target products, a process that entails substantial chemical inputs (Fig. 3A). Taking Li extraction as an example, hydrochloric acid is initially used to adjust the brine’s pH, followed by specific organic solvents to remove BO_3_^3−^. Lime is then introduced to precipitate Mg^2+^ and Ca^2+^ ions. Subsequently, sodium carbonate is added to precipitate Li_2_CO_3_ from the Li-rich brine. If LiOH is the desired product, an additional reaction with lime is required to convert Li_2_CO_3_ into LiOH. For brines with a Mg/Li mass ratio of 10, producing 1 ton of Li_2_CO_3_ requires at least 1.4 tons of sodium carbonate and 4.3 tons of quicklime for magnesium removal, not including other chemical consumption. For brines with higher Mg/Li ratios, chemical consumption increases sharply, potentially rendering the process economically unfeasible. Therefore, developing chemical-free methods for target minerals is appealing and essential for the sustainability of brine mining processes (*42*). ISE technologies with advanced extraction strategies, such as sequential crystallization (Fig. 3D) (*39*, *105*), membrane separation (Fig. 3E) (*106*, *107*), and selective adsorption (Fig. 3F) (*108*–*118*), offer promising approaches to reduce chemical uses in the mining process. In principle, evaporative flow drives water molecules and ions through the evaporator as water is released. This flow promotes interactions with the evaporator’s structure, enabling selective separation through specific nano/microstructure designs. It has been demonstrated that as the brine passes through cellulose strings with porous and tortuous channels, sequential and spatial separation of ions like Li^+^ and Na^+^ occurs. In this setup, NaCl crystallizes in the lower sections, while LiCl accumulates near the top of the strings, offering a refined and efficient approach for target mineral separation (*39*).

Beyond the spatial separation that primarily relies on ions’ solubility difference, solar evaporators can also facilitate the target mineral extraction through their combination with membrane separation structures (*106*, *107*). This innovative design allows the membrane structure to selectively filter desired constituents, ensuring that only the target minerals can enter the evaporator while effectively removing undesirable components. Concurrently, solar evaporation generates transpiration pressure to drive the influx of target minerals from the brine into the evaporator. Recently, Song *et al.* (*107*) introduced an evaporation-based device with a sophisticated three-layer design, comprising a nanofiltration membrane at the base for selective ion separation, a middle silica layer that serves as Li^+^ storage, and a top photothermal layer to drive the evaporation. This device demonstrated feasibility through series expansion and seamless integration with evaporation ponds, showcasing the potential of chemical-free mineral extractions.

In addition, embedding selective sorbents or incorporating functional binding sites into the evaporator represents another effective strategy for mineral extraction. In such a design, evaporation-driven flow drives the rapid transport of minerals toward the sorbent, promoting selective and rapid capture. Meanwhile, localized photothermal heat can further increase the adsorption capacity and kinetics. For selective Li^+^ extraction, promising progress has been made by incorporating Li^+^-selective adsorbents (such as Li_4_Ti_5_O_12_ and Li_1.6_Mn_1.6_O_4_) into photothermal structures. The combined photothermal and evaporative effects have been shown to markedly enhance both adsorption kinetics and capacity of these materials (*108*, *109*).

Similar strategies have also been used to extract other ions such as uranyl (UO_2_^2+^). For example, incorporating programmable DNA into a photothermal hydrogel allows for the selective adsorption of uranium ions (*111*). This uranyl-specific DNA hydrogel exhibited a high capture capacity of 5.7 g g^−1^ for UO_2_^2+^, along with excellent selectivity, showing a 10.4-fold preference over vanadium. Beyond this DNA-based hydrogel, uranyl-selective sorbents including glyphosine-modified chitosan (*112*) and poly(amidoxime) sorbents (*113*, *115*–*117*) have been combined with photothermal materials to achieve selective UO_2_^2+^ extraction. In addition, grafting hyperbranched amidoxime groups onto photothermal fibers has also demonstrated enhanced affinity toward UO_2_^2+^ (*114*). Selective extraction of cesium ions (Cs^+^) by integrating phase-change material has also been reported (*118*). By embedding with crown ether–functionalized phase-change microcapsules, the system achieved a high evaporation rate of 1.43 kg m^−2^ hour^−1^ and a Cs^+^ adsorption capacity of 32.6 mg g^−1^ (*118*).

These advancements highlight the great potential of ISE technology in facilitating selective mineral extraction, particularly when combined with other technologies. Although research in this field is still in its early stages, ongoing exploration and optimization are poised to drive substantial progress and ultimately transform the landscape of mineral recovery practices.

### ISE for beneficial water harvesting

During the evaporative concentration process, a great amount of fresh water is lost as vapor. According to SQM’s 2023 annual report (*119*), the company produced around 210,000 tons of Li_2_CO_3_ annually, indicating that 37.6 million m^3^ of water was lost as vapor during the concentration process, calculated on the basis of a Li^+^ concentration of 1500 ppm (parts per million) in brine and a recovery efficiency of 70% (*44*, *92*). While studies suggest that the Atacama basin, where the brine is located, is not hydrogeologically connected to the groundwater coming from the Andes that is used by communities (*120*), and the company was operated within its water permits, there is a lost opportunity, as if this evaporated water could be recovered, it can provide enough daily water use for ~0.5 million people, assuming a daily water consumption of 200 liters per person in Chile (*121*). Such recovery would offer tremendous benefits to both local communities and industries, particularly in arid and semiarid regions where brine ponds are typically located and water scarcity is severe. In addition to water loss during evaporation, fresh water is consumed during subsequent extraction and purification processes. Reports indicate that producing 1 ton of battery-grade Li_2_CO_3_ requires 22.5 m^3^ of fresh water in Salar de Atacama and 50 m^3^ in Salar de Olaroz (*44*, *92*). Therefore, many mining companies drill wells to extract groundwater to secure a reliable freshwater supply (*92*). Moreover, despite the many benefits direct lithium extraction technologies (DLEs) will bring to the industry, dominant DLE processes such as ion exchange and solvent extraction have been reported to consume more than 500 m^3^ per ton of LCE, many times higher than current ponds (*44*). As the global demand for critical minerals continues to surge, the scale of brine mining is expanding, and water will continue to be a major concern associated with the industry.

Solar evaporators rapidly generate vapor under sunlight. Once operated in an enclosed chamber, the generated vapor condenses upon reaching 100% relative humidity, facilitating vapor recovery and yielding fresh water (Fig. 3G) (*109*, *122*–*124*). Integrating ISE with advanced vapor condensation systems thus offers a promising pathway for mitigating water loss and achieving simultaneous mineral extraction and freshwater harvesting. However, realizing this potential hinges on the codevelopment of efficient vapor recovery systems, which have been advanced in recent years in conjunction with ISE desalination systems. In the absence of water harvesting systems, the enhanced evaporation driven by ISE may instead lead to accelerated water loss rather than recovery.

A floating solar still was reported to achieve a daily freshwater production of ~2.5 liters m^−2^, corresponding to a solar-to-water efficiency of ~20% (*59*). Through thermal management to reduce heat dissipation, daily water production can be further improved to 5 liters m^−2^ with an efficiency of ~40% (*55*). In addition, the current condensation chamber is made from transparent glass or plastic panels, which usually exhibit low thermal conductivity that impedes the release of condensation heat and consequently affects vapor recovery efficiency. To further improve the freshwater production, researchers have proposed inverted structures based on high-conductivity metals (*125*–*127*), achieving a solar-to-water efficiency of ~70% (*125*). In addition, by recycling the condensation heat instead of dissipating it (Fig. 3H), multistage evaporators can even achieve an impressive solar-to-water efficiency exceeding 300% in brine with a salinity of 20 wt % (Fig. 3I) (*128*). Despite notable advancements in vapor condensation and freshwater production, it remains very challenging to implement water harvesting during evaporation of solar brine ponds. The expansive size of these ponds complicates the installation of efficient water recovery systems. High salinity levels can cause rapid equipment corrosion and fouling, reducing system longevity and effectiveness. In addition, transporting and storing harvested water from remote pond locations require substantial infrastructure, posing logistic and economic challenges. Moreover, introducing water harvesting mechanisms may interfere with the natural evaporation process, potentially decreasing the overall efficiency of mineral concentration in the brine. Addressing these obstacles necessitates innovative engineering solutions and investment to make water harvesting in solar brine ponds a viable and sustainable practice.

### ISE for technoeconomic transformation

Economic feasibility is a key determinant for translating new technologies into industrial practice. Traditional solar evaporation ponds remain the most widely adopted approach for brine mining, largely due to their cost-effectiveness, with capital expenditures (CAPEX) typically ranging from $23,000 to $34,000 per ton of LCE (*129*). In contrast, emerging DLE technologies such as membrane separation, adsorption, and ion exchange often carry substantially higher CAPEX ($45,000 to $80,000 per ton of LCE) (*129*). Given that ISE can be integrated into existing ponds without major process or energy modifications, its deployment is not expected to markedly increase operating expense (OPEX). On the contrary, by improving evaporation rates, facilitating heat management, and enabling selective mineral capture, ISE may contribute to streamlining the operation and reduce OPEX.

The primary cost consideration for ISE deployment lies in the evaporator device and materials. A recent study has suggested that the material cost of scalable ISE evaporators can be as low as ~$0.5 per square meter (*130*), but it only represents a small fraction of total device cost, although large-scale cost data remain scarce. To illustrate ISE’s economic potential, for example, a 1-ha solar evaporation pond typically yields 50 to 70 tons of LCE per year in Atacama. A stabilized price of $15,000 to $20,000 per ton translates to $750,000 to $1,400,000 in annual revenue. While field demonstration showed a 40 to 122% increase in evaporation rates using Lilypad evaporators (*104*), lab-scale evaporators reported improvement ranging from two to four times (*131*). Because of the wide range of operation scales, system architecture, and performance under different brine conditions, our preliminary technoeconomic analysis used twofold enhancement as an overall relative conservative estimation. Under this scenario, the additional LCE output would generate an additional million-dollar range of revenue per hectare each year.

In addition to the CAPEX and OPEX, the long-term viability of mining practice is critically shaped by its environmental impacts, including energy, water, land, and chemical use. While there are limited data available to directly compare ISE technologies on a scale with solar evaporation ponds and DLE, and such a comparison is beyond the focus of this study, key distinctions in such aspects are worth noting, so further analysis can be carried out when more data become available. Compared to DLE technologies, which are energy, water, and chemical intensive as previously discussed, the ISE approach largely relies on passive solar energy, resulting in minimal energy requirements. Life cycle assessments indicate that DLE processes can emit up to 22 tons of CO_2_ equivalent per ton of LCE depending on the electricity source. In contrast, traditional solar ponds emit ~6.2 tons of CO_2_ equivalent per ton of LCE (*132*). The energy footprint for ISE should be similar to that of traditional solar ponds, although additional electricity will be used for deployment, periodic cleaning, and harvesting. Furthermore, ISE does not use additional chemicals and reduces land use by 50% or more compared to conventional ponds. The impact on water is more complex depending on whether an ISE technology also recovers fresh water. Current simple “Lilypad-type” devices do not recover fresh water, but it is conceivable that next-generation ISE devices could be designed to not only accelerate evaporation but also capture the resulting vapor. If such advanced technologies prove to be economically viable, they would offer substantial environmental benefits across all key metrics: energy, land, water, and chemical use.

While these preliminary estimates on cost-effectiveness and environmental performance await validation by commercial-scale projects, they underscore ISE’s strong potential as a transformative, low-impact upgrade to conventional solar pond operations. Field trials will be essential to confirm its true economic viability, long-term performance, and robustness under diverse environmental conditions.

### Outlook: challenges and opportunities

**Fig. 4.**
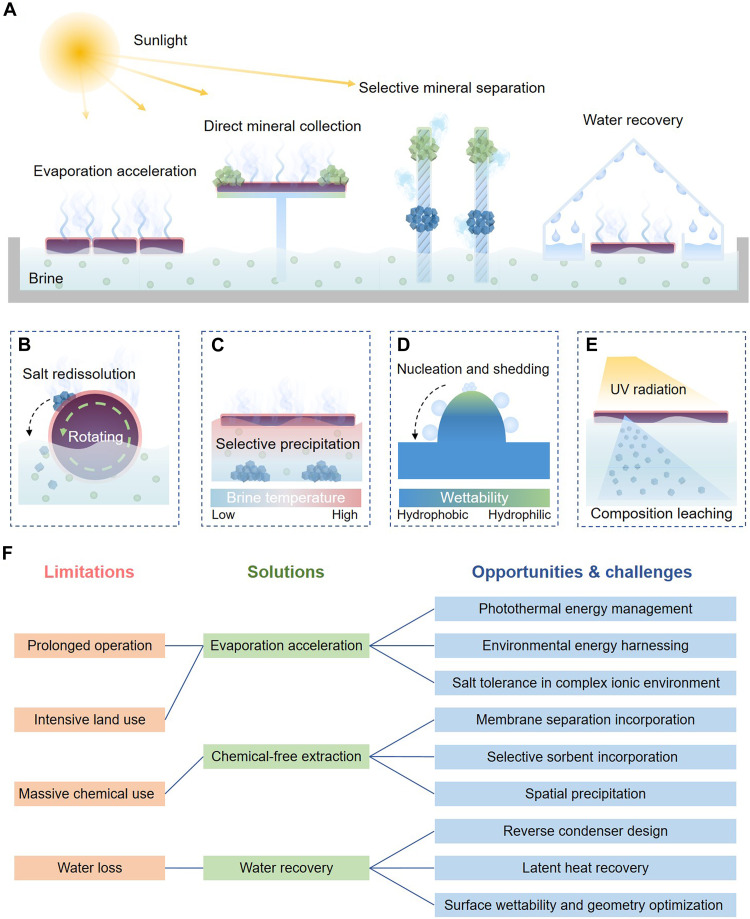
Future brine pond operation with the integration of ISE technology. (**A**) Vision for integrating ISE into an evaporation pond to accelerate evaporation, enable direct mineral collection, and facilitate selective mineral extraction and freshwater harvesting. (**B**) Demonstration of a self-rotating evaporator that is sensitive to weight balance. (**C**) Schematic of temperature gradient–enhanced fractional precipitation realized by evaporator deployment. (**D**) Bioinspired surface with altering wettability for enhanced condensate collection. (**E**) Demonstration of composition leaching during prolonged solar exposure. (**F**) Limitations, solutions, opportunities, and challenges in advancing ISE for brine mining.

This perspective review highlights the potential of ISE technology to transform the evaporation-based brine mining process (Fig. 4A). By driving evaporation at the air-liquid interface through engineered photothermal materials and robust heat management, ISE can reduce processing time, curtail water losses, lower chemical requirements, and help limit the future land expansion associated with brine mining. However, moving from laboratory innovations to practical applications involves a range of challenges and opportunities (Fig. 4, B to F), necessitating more in-depth scientific and engineering efforts. There remain many challenges and opportunities for ISE to adapt to complex ionic environments, achieve multifunctional separation, enable water collection, and eventually transition from laboratory prototypes to industrial deployment.

### Adaptability in a complex brine environment

ISE accelerates evaporation to overcome the prolonged operation and large footprint required by conventional ponds, but maintaining stable, high evaporation rates in high-salinity brines is challenging. Although numerous salt-resistant designs work well under seawater-like conditions (~3.5 wt % salinity), many brines exceed 5 wt % and can reach up to 35 wt % or saturation. Under these extreme conditions, the salt backflow mechanism that prevents salt buildup diminishes, so backflow-based evaporators may not be suitable under such conditions. Instead, strategies involving Janus structures or contactless configurations hold good promise in high-salinity scenarios. By preventing direct brine intrusion or physically separating the photothermal surface from the brine, these designs reduce the risk of fouling and preserve high evaporation rates. The innovative nano/microscale structures and system-level architectures capable of actively shedding crystallized salts are especially desired.

While many evaporators exhibit excellent stability in NaCl solutions, commonly used for salt-tolerant performance evaluation, their effectiveness in real brines remains largely underexplored. Real brines can contain diverse ions (e.g., Mg^2+^, Ca^2+^, and SO_4_^2−^) that form dense crystalline deposits, especially when multiple salts coprecipitate. Compared to simpler NaCl crystals, these deposits often adhere strongly to evaporator surfaces, clogging pores and fouling wicking channels. Given that brine is geographically diverse and the composition changes over time, evaporators must tolerate shifting salinity profiles and varied ionic ratios. Advanced structure designs, ranging from specialized coatings (*71*, *132*) to self-cleaning architectures (*133*–*136*), can help mitigate these complications and maintain stable performance over extended operation. For instance, an Al_2_O_3_ and carbon black coating on copper foam can create a superhydrophobic photothermal layer (*71*). This specialized coating, acting as a barrier, can effectively prevent brine intrusion, thereby allowing the evaporator to operate stably in saturated brines with multiple components (*71*, *132*). In addition, self-rotating architectures offer an innovative self-cleaning strategy (Fig. 4B) (*133*–*136*). These designs rely on the evaporator’s responsiveness to weight balance. When salt crystals accumulate on the evaporator surface, the resultant asymmetry in mass distribution causes the spherical or cylindrical architecture to automatically rotate. This motion facilitates the redissolution or detachment of crystals, effectively cleaning and refreshing the evaporative surface and enabling long-term stable operation even in high-salinity brines (*135*).

### Multifunctional evaporators for selective mineral extraction

Beyond high evaporation rates, selective mineral recovery is a crucial goal in brine mining. Traditional ponds rely on multistage concentration and heavy chemical usage to precipitate target minerals. ISE systems, by contrast, can potentially reduce chemical demands and simplify processes through multifunctional designs. These multifunctional evaporators are engineered not only for efficient water evaporation but also for selective mineral recovery, leveraging intrinsic differences among the minerals, such as size, charge, solubility, and specific binding affinities. During the evaporation, evaporative flow drives the directional transport of minerals, increasing their interaction with the evaporator and creating opportunities for selective mineral extraction. For example, coupling ISE with size- or charge-selective membranes allows for ion-specific separation, guiding valuable elements like Li^+^ or Mg^2+^ toward distinct collection pathways (*106*, *107*). In parallel, incorporating specialized sorbents (e.g., functional DNA units and covalent organic framework) into the evaporator harnesses localized concentration gradients, promoting high-efficiency adsorption of target ions (*111*, *137*). In addition to the opportunities created by evaporative flow, photothermal effects can generate localized microenvironments characterized by elevated temperature. By exploiting differences in ion solubility under different temperature conditions, these gradients present promising opportunities for controlling selective precipitation and crystallization processes (Fig. 4C) (*104*). Such processes can usually be described using phase diagrams and thermochemical modeling (*50*), correlating temperature and ion concentration to guide the stepwise recovery of valuable minerals. Leveraging the synergy between ISE and various extraction technologies holds immense potential for advancing selective mineral extraction. However, research in this area remains relatively nascent, requiring further efforts to address challenges such as optimizing technology integration, improving selectivity, and enhancing efficiency.

### Optimized structures and systems for water collection

Vapor recovery offers a promising approach to mitigate the water loss inherent in evaporation ponds. ISE systems can theoretically recover a share of this vapor, yet practical water collection often lags behind laboratory efficiencies. This issue originates from inefficiencies of vapor condensation; meanwhile, as vapor accumulates in an enclosed or semienclosed space, rising pressure slows additional evaporation. Vapor recovery involves a sequence of nucleation, coalescence, and droplet shedding, with overall efficiency governed by heat and mass transfer processes (*138*). To achieve rapid condensation, condensation heat must be dissipated efficiently to ensure that the condensation surface remains sufficiently cool, maintaining optimal conditions for the process to continue. Innovative designs, such as reverse condensation architectures, have made progress in this regard (*125*, *126*). In addition, incorporating radiative cooling materials to passively dissipate heat through thermal radiation also offers another attractive approach for enhancing condensation efficiency (*139*, *140*).

As condensed droplets grow and coalesce, their rapid shedding becomes essential. This is because the attached water film due to its low thermal conductivity (~0.6 W m^−1^ K^−1^) (*138*) impedes latent heat dissipation and hinders subsequent condensation. Strategies to enhance droplet shedding can be optimized by tailoring surface wettability and geometry (*141*). Insights drawn from nature offer valuable guidance for designing surfaces with enhanced condensation performance. For instance, beetles in arid environments have evolved surfaces for fog collection featuring alternating hydrophilic and hydrophobic regions (Fig. 4D) (*142*). Similarly, the spines of cacti exhibit a unique conical shape that can facilitate droplet collection through Laplace pressure gradients (*143*). These bioinspired strategies for efficient vapor collection can play a critical role in improving water recovery. Moreover, by mitigating vapor pressure buildup within sealed systems, they help sustain evaporation rates, thereby enhancing the overall effectiveness of ISE in brine mining processes.

### Awareness and management of potential environmental risks

ISE is widely recognized as an environmentally friendly approach. However, as the technology advances toward large-scale implementation, a comprehensive understanding of its long-term environmental impact becomes increasingly critical, but it remains insufficiently addressed in current studies.

One critical concern lies in the stability of materials used in evaporators. Brines are characterized by high salinity and complex ionic compositions, often exhibiting strong corrosive effects, particularly on evaporators containing metallic components (*144*). The corrosion rate of steel in saline water can exceed 0.6 mm per year depending on the pH, temperature, and salinity degree (*145*). Furthermore, prolonged exposure to solar radiation can also impose stress on polymeric structures, leading to structural embrittlement and the potential release of compositions (Fig. 4E) (*146*). For example, after 1000 hours of exposure to ultraviolet (UV) irradiation, the surface roughness of epoxy resin decreases by 25%, and its weight is reduced by ~0.03% because of the loss of volatile compositions (*147*). Over extended operational periods, such degradation and corrosion processes may result in the leaching of toxic substances, thereby posing latent risks to the surrounding environment.

In addition to the risks associated with material degradation, the ecological implications of extensive ISE deployment merit equal attention. Salt lakes often support unique ecosystems that serve as habitats or stopover sites for bird species (*148*). For example, the Great Salt Lake provides a critical habitat for more than 230 bird species, including an estimated 8 to 10 million migratory birds annually (*148*). Widespread coverage of evaporators may alter the microclimatic conditions and limit the access to the open water surface, thereby potentially disrupting local biodiversity dynamics (*149*).

As an emerging technology for brine mining, ISE currently lacks clear guidelines for large-scale deployment in the real-world environment. We therefore emphasize the need for future research to assess its ecological implications comprehensively. Key challenges include improving the stability of photothermal materials under harsh brine and UV conditions and establishing real-time environmental monitoring systems to ensure safe operation. Moreover, initial deployment should prioritize small-scale pilot studies, such as experimental integration with existing solar ponds, to facilitate close environmental observation and feedback. Such gradual scaling is essential for establishing ecologically responsible deployment protocols in future applications.

### The need of system engineering and beyond for commercialization

Beyond its great promises, scaling ISE from bench-scale prototypes to multihectare brine fields demands a careful balance among efficiency, robustness, and affordability. Although some high-performance designs rely on expensive materials or intricate manufacturing, transitioning from laboratory investigations to industrial brine mining requires thoughtful system engineering that emphasizes stability, scalability, and cost efficiency. Durability in this context spans resistance to complex ionic environments and structural integrity under high temperatures and prolonged UV exposure. Widespread deployment of laboratory prototypes remains a notable obstacle, necessitating scalable manufacturing approaches such as roll-to-roll fabrication, 3D printing, and other cost-effective production methods. Modular designs and easily assembled evaporator structures are particularly valuable for ensuring adaptability across diverse operational scenarios. Moreover, material innovation should prioritize reducing dependence on rare or expensive components. Integrating biobased or recycled materials offers a promising pathway to achieving cost-effective yet high-performing systems.

Field trials are crucial to validate critical performance metrics—evaporation rate, salt tolerance, water yield, and operational longevity—under actual brine compositions and varying climatic conditions. Pilot data also inform cleaning protocols, salt harvesting strategies, and brine feed requirements. In parallel, machine learning or sensor-based optimization can anticipate fouling events, fine-tune brine flows, and maintain consistent productivity. History has also shown that regulatory frameworks and community acceptance are pivotal for real-world success. Demonstrating that ISE minimizes the need for future land expansion or reducing existing footprint, reduces chemical usage, and recovers water can strengthen the social license to operate and simplify permitting processes. By engaging proactively with local stakeholders and conducting transparent impact assessments, mining initiatives can better align with environmental stewardship and community well-being.
